# Safety and immunogenicity after a 30-month boost of a subtype C ALVAC-HIV (vCP2438) vaccine prime plus bivalent subtype C gp120/MF59 vaccine boost (HVTN 100): A phase 1–2 randomized double-blind placebo-controlled trial

**DOI:** 10.1371/journal.pgph.0003319

**Published:** 2024-09-20

**Authors:** Vimla Naicker, Fatima Laher, Linda-Gail Bekker, Kelly E. Seaton, Mary Allen, Stephen De Rosa, Nicole L. Yates, Nonhlanhla N. Mkhize, Kevin Saunders, Jack Heptinstall, Mookho Malahleha, Kathryn Mngadi, Brodie Daniels, Craig Innes, Chenchen Yu, Tandile Modise, Valerie Bekker, Nicole Grunenberg, Briana Furch, Maurine D. Miner, Sanjay Phogat, Carlos A. Diazgranados, Sanjay Gurunathan, Marguerite Koutsoukos, Olivier Van Der Meeren, Alison C. Roxby, Guido Ferrari, Lynn Morris, David Montefiori, M. Juliana McElrath, Georgia D. Tomaras, Zoe Moodie

**Affiliations:** 1 South African Medical Research Council, Durban, South Africa; 2 Faculty of Health Sciences, Perinatal HIV Research Unit, University of the Witwatersrand, Johannesburg, South Africa; 3 Desmond Tutu HIV Centre, University of Cape Town, Cape Town, South Africa; 4 Departments of Surgery and Integrative Immunobiology, Duke Human Vaccine Institute, Durham, North Carolina, United States of America; 5 Vaccine Research Program, Division of AIDS, National Institute of Allergy and Infectious Diseases, National Institutes of Health, Bethesda, Maryland, United States of America; 6 Vaccine and Infectious Disease Division, Fred Hutchinson Cancer Research Center, Seattle, Washington, United States of America; 7 National Institute for Communicable Diseases, National Health Laboratory Service, Johannesburg, South Africa; 8 Faculty of Health Sciences, SAMRC Antibody Immunity Research Unit, University of the Witwatersrand, Johannesburg, South Africa; 9 Synergy Biomed Research Institute, East London, South Africa; 10 Centre for the AIDS Programme of Research in South Africa, Durban, South Africa; 11 Aurum Institute, Klerksdorp Research Centre, Klerksdorp, South Africa; 12 Sanofi Pasteur, Swiftwater, Pennsylvania, United States of America; 13 GSK, Wavre, Belgium; 14 Previously GSK, Rixensart, Belgium; 15 University of Washington Departments of Medicine and Global Health, Seattle, Washington, United States of America; 16 Department of Surgery, Center for Human System Immunology, Duke University School of Medicine, Durham, North Carolina, United States of America; Technical University of Kenya, KENYA

## Abstract

Induction of broad, durable immune responses is a challenge in HIV vaccine development. HVTN 100 Part A administered subtype C-containing ALVAC-HIV at months 0 and 1, and ALVAC-HIV with bivalent subtype C gp120/MF59 at months 3, 6 and 12. As IgG binding antibody and T-cell responses were similar or greater at month 12.5 vs. month 6.5, but waned by month 18, we investigated vaccine-elicited immune responses after a month 30 boost in this study, HVTN 100 Part B. From 13 September 2017 to 7 August 2018, a subgroup of vaccinees was randomized to receive intramuscular injections of ALVAC+gp120/MF59 (n = 32) or gp120/MF59 alone (n = 31) and a subgroup of placebo recipients was administered placebo (n = 7) at month 30. Primary outcomes were safety, IgG binding antibodies (bAbs) to vaccine-specific and V1V2 Env proteins and vaccine-specific CD4+ T cells at month 30.5. Secondary outcomes included neutralizing and antibody dependent cellular cytotoxicity functions and durability at months 30 and 36. Both vaccine groups had an acceptable safety profile. There were no statistically significant differences in the occurrence or level of IgG bAbs between the vaccine boost groups for any vaccine-specific or V1V2 antigens. IgG responses were higher to vaccine-matched gp120 than to V1V2. The booster vaccination restored the magnitude-breadth IgG bAb response to V1V2 antigens at month 30.5. However, it rapidly waned by month 36. CD4+ T-cell response rates to the 3 vaccine-matched Env antigens for the combined vaccine groups ranged from 37% at month 30, boosted to as high as 91% at month 30.5, and waned by month 36 to as low as 44%, with no significant differences between the vaccine boost groups. Because these responses waned after 6 months, additional strategies may be needed to maintain the durability of prime-boost vaccine regimens and to generate these or other immune responses that confer protection.

**Trial registration:** South African National Clinical Trials Register (SANCTR number: DOH—27-0215-4796) and ClinicalTrials.gov (NCT02404311).

## Introduction

Effective HIV vaccines require robust and sustained immune responses. RV144, the only HIV vaccine regimen trial that has shown any efficacy to date, demonstrated waning efficacy coinciding with waning immune responses after a heterologous prime-boost strategy given at months 0, 1, 3 and 6 [1]. The timing of vaccine doses may affect immune responses. Animal studies suggest that shortening the interval between boosters, for example to two weeks apart, may result in a faster time to achieving protection, but could unfavorably affect the formation of stable long-term memory T cells [2]. In addition to the question of optimal dose timing, it is also of interest to investigate whether waning responses could be mitigated with serial boosting to maintain critical responses above a threshold associated with efficacy.

RV305 and RV306 were clinical trials that followed the partially efficacious RV144 HIV vaccine trial. RV305 was a randomized, double-blind placebo-controlled trial that evaluated the immunogenicity of late boost strategies in 162 RV144 vaccine recipients who did not acquire HIV-1 and who had completed the full RV144 immunization series [3]. The late boost was after 6–8 years with RV144 immunogens (ALVAC-HIV alone, AIDSVAX B/E gp120 alone, or ALVAC-HIV + AIDSVAX B/E gp120). The results showed that the ALVAC vaccine component contributed to improved breadth, function, and durability of vaccine-elicited antibody responses [3,4]. The RV306 trial enrolled and randomized 360 healthy volunteers in Thailand to receive the RV144 priming series over 6 months, followed by no boost, or a boost with AIDSVAX B/E alone, or a boost with AIDSVAX B/E plus ALVAC at month 12, or a boost with both vaccines at month 15 or 18. The results showed that the Fc-mediated effector functions were increased 2 weeks after the additional boost at week 48 compared to 2 weeks after completion of the RV144 vaccination regimen but the responses were not durable [5]. In HVTN 100 Part B, we used a more compressed vaccination schedule, which aimed to improve the durability of the immune response compared to RV306.

In HVTN 100, a Phase 1–2 trial of a pox-protein vaccine regimen tailored for the sub-Saharan African region, participants were vaccinated at the same time points as RV144, with an additional vaccination of ALVAC-HIV+gp120/MF59 at month 12 [6]. This resulted in immune response extension [7], however responses waned by month 18, contributing to a decision to add a vaccination at month 18 to HVTN 702, the Phase 2b-3 trial of the same regimen that ultimately showed no efficacy [8].

The results from the RV305 and RV306 trials suggest that boosting HVTN 100 trial participants at month 30 with bivalent subtype C gp120/MF59 with and without ALVAC-HIV (vCP2438) may restore immune responses, especially those correlated with decreased HIV acquisition risk in RV144 [4].

Our study investigated how each vaccine component—all components versus adjuvanted protein only—contributes to the immune response to a late booster of the subtype C-adapted ALVAC/gp120 HIV vaccine regimen. We demonstrate the effect of late booster vaccinations on safety and immune responses. We characterized cellular, binding and neutralizing antibody, and functional immune responses elicited by different booster vaccinations at 30 months after the first vaccination in HVTN 100 participants.

## Methods

### Trial design

The HVTN 100 trial has been previously described [6]. In summary, it was a Phase 1–2 randomized, double-blind, placebo-controlled clinical trial of subtype C ALVAC-HIV (vCP2438) and bivalent subtype C gp120/MF59 vaccines administered at months 0, 1, 3, 6, and 12 (Table 1). Six trial sites in South Africa enrolled 252 eligible participants: healthy adults living without HIV, aged 18–40 at low vulnerability for HIV acquisition.

**Table 1:** HVTN 100 Part A and B schema.

Group	N	**Primary vaccine regimen Part A**				**Booster**
		Month 0	Month 1	Month 3	Month 6	Month 12
1	210	ALVAC-HIV (vCP2438)	ALVAC-HIV (vCP2438)	ALVAC-HIV (vCP2438) + Bivalent Subtype C gp120/MF59	ALVAC-HIV (vCP2438) + Bivalent Subtype C gp120/MF59	ALVAC-HIV (vCP2438) + Bivalent Subtype C gp120/MF59
2	42	Placebo	Placebo	Placebo + Placebo	Placebo + Placebo	Placebo + Placebo
Total	252					
				**Booster** **Part B**		
Part A Group		Part B Group^†^	N	Month 30		
1		1a	32	ALVAC-HIV (vCP2438) + Bivalent Subtype C gp120/MF59		
		1b	31	Placebo + Bivalent Subtype C gp120/MF59		
2		2	7	Placebo + Placebo		
Total 70						

**Fig 1:**
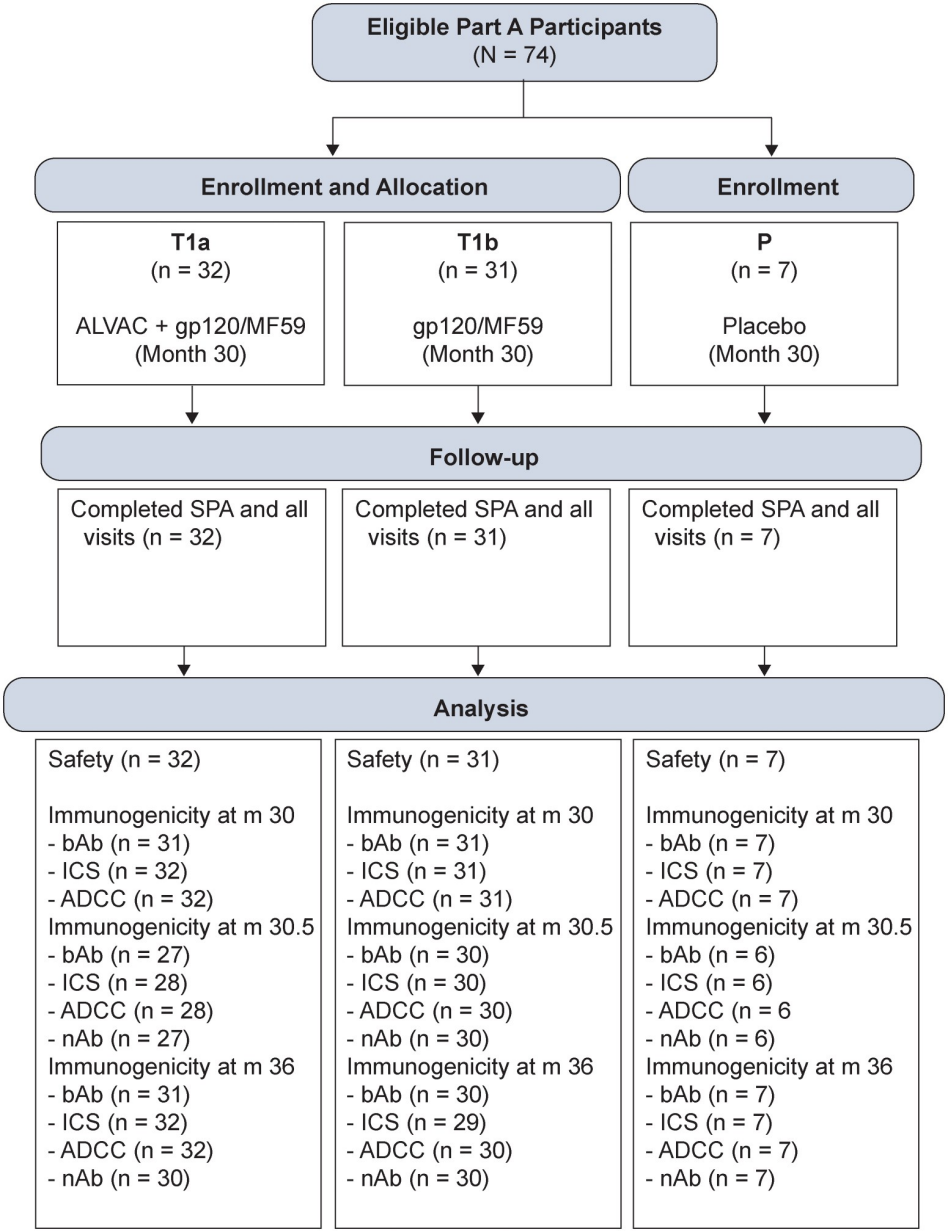
CONSORT diagram. SPA = study product administration; bAb = binding antibody; ICS = intracellular cytokine staining; ADCC = antibody dependent cellular cytotoxicity; nAb = neutralizing antibody.

In Part A, the trial followed 252 participants for safety and immunogenicity until month 18. The extension of the trial into HVTN 100 Part B maintained the double-blinded, placebo-controlled design with planned enrollment of n = 70 participants. Participants from HVTN 100 Part A who had neither met discontinuation of vaccination criteria nor criteria for termination from the study and agreed to provision of mucosal samples as per protocol were eligible. All participants provided written informed consent. Of the 70 participants enrolled in Part B, 63 participants had received vaccine in Part A. Of these, 32 were randomized to ALVAC+gp120/MF59, 31 were randomized to gp120/MF59 alone and seven participants who received placebo in Part A received placebo again in Part B (Fig 1). These participants received vaccinations at month 30 and were followed until month 36 (Table 1).

Randomization sequences for Part B vaccine groups were obtained by computer-generated block randomization through a Web-based randomization system to achieve balance across both vaccine groups. Participants who received placebo in Part A also received placebo in Part B.

### Ethics statement

The research ethics committees of the University of the Witwatersrand, the University of Cape Town, the University of KwaZulu-Natal, and the South African Medical Research Council approved the study. The trial was overseen by the NIAID DSMB. HVTN 100 was registered with the South African National Clinical Trials Register (SANCTR number: DOH—27-0215-4796) and ClinicalTrials.gov (NCT02404311).

### Trial objectives

A primary objective for HVTN 100 Part B was to evaluate the safety and tolerability of bivalent subtype C gp120/MF59 and of ALVAC-HIV (vCP2438) + bivalent subtype C gp120/MF59 when given as boosts in participants previously vaccinated in HVTN 100 Part A. The primary endpoints included the frequency of severe local and systemic reactogenicity signs and symptoms and the primary immunogenicity objective was to evaluate cellular and binding antibody responses at two weeks following vaccination with bivalent subtype C gp120/MF59 or ALVAC-HIV (vCP2438) + bivalent subtype C gp120/MF59 when given as boosts at month 30 in participants previously vaccinated in Part A. The primary immunogenicity outcomes were prevalence and level of vaccine-induced IgG antibody binding to envelope (Env) proteins, vaccine-induced IgG antibody binding to V1V2-scaffolded Env proteins and vaccine-induced CD4+ T-cell responses to the HIV proteins included in the vaccine at month 30.5. HVTN 100 Part B was designed while the HVTN 702 study was still ongoing and the lack of efficacy not yet known.

HVTN 100 Part B secondary objectives included evaluation of the durability of immune responses at months 30 and 36 and further characterization of the HIV-1 specific immunogenicity of ALVAC-HIV (vCP2438) vs. ALVAC-HIV (vCP2438) + bivalent subtype C gp120/MF59 at 2 weeks following vaccination at month 30.

### Study products and routes of administration

The study products were ALVAC-HIV (vCP2438) and MF59-adjuvanted bivalent subtype C gp120. ALVAC-HIV (vCP2438) (viral titer nominal dose of 10^6^ to 10^8^ 50% cell culture infectious dose) expressed the Env gp120 of the subtype C ZM96.C strain with the gp41 transmembrane sequence of the subtype B LAI strain, as well as Gag and protease from the subtype B LAI strain. Bivalent subtype C gp120 was a combination of 100 μg each of the subtype C Env gp120 of the TV1.C and 1086.C strains. Placebo for ALVAC-HIV was a mixture of virus stabilizer and freeze-drying medium reconstituted with 0.4% NaCl, and placebo for the bivalent subtype C gp120 was sodium chloride for injection.

### Safety monitoring

Participants were observed at the clinical research sites for 30 minutes post vaccination and completed a post-vaccination symptom log for 3 days to track local and systemic reactogenicity signs and symptoms.

Safety assessment symptoms of pain, tenderness, erythema, and induration at the injection site were assessed as local reactogenicity, and malaise and/or fatigue, myalgia, headache, nausea, vomiting, chills, arthralgia and elevated temperature were assessed as systemic reactogenicity if the onset date was within 72 hours following vaccination. Adverse events (AEs) were graded using version 2.0 of the DAIDS Table for Grading the Severity of Adult and Pediatric Adverse Events (November 2014). The safety laboratory parameters included: white blood cells, neutrophils, lymphocytes, hemoglobin, platelets, alanine aminotransferase, aspartate aminotransferase, alkaline phosphate, and creatinine.

### Laboratory immunogenicity assays

Cellular and antibody responses were evaluated at months 30 (18 months post 5^th^ vaccination), 30.5 (2 weeks post 6^th^ vaccination) and 36 (6 months post 6^th^ vaccination).

The frequency, magnitude and breadth of IgG binding antibody (bAb) responses were measured by the HIV-1 binding antibody multiplex assay (BAMA) in serum specimens as previously described [6]. Serum HIV-1-specific IgG (1:50 dilution) responses against antigens listed (S1 Table) were measured on a Bio-Plex instrument (Bio-Rad) using a standardized custom HIV-1 Luminex assay [9–13]. Antibody-dependent cell-medicated cytotoxicity (ADCC) antibody responses were measured using GranToxiLux (GTL) assays and a luciferase assay (S1 Text). The neutralizing antibody (nAb) assay was performed with Env-pseudotyped viruses in TZM-bl cells as previously described [6]. Intracellular cytokine staining (ICS) was performed to measure HIV-specific CD4+ and CD8+ T cells expressing IFN-γ, IL-2, or CD40L as previously described [6], but using a 17-color staining panel. CD4 and CD8 T-cell responses were classified according to 4 different phenotypes: naïve; central memory; effector memory and terminally differentiated T-cells, depending on the presence of CCR7 and/or CD45RA receptors. Information about antigens used in all assays is listed in S1 Table.

### Statistical analyses

All enrolled participants contributed to the safety analysis. Participants with samples available who met assay-specific quality control criteria were included in the immunogenicity analysis; participants who had an HIV-1 positive test prior to the immunogenicity blood draw were excluded. Enrollment into Part B was contingent on per-protocol receipt of all Part A immunizations.

The sample size for the Part B groups provides sufficient precision to address the primary objectives to characterize safety and immunogenicity. If none of the 60 participants boosted in Part B (n = 60 total) experience a safety event, the 95% 2-sided upper confidence bound for the true rate of such events in the total vaccinated population is 6.02%. Similarly, the sample size allows the prevalence of positive immunogenicity responses in each boost group to be estimated with reasonable precision, assuming 15% missing data (n = 26 each). For example, 50% prevalence has 95%CI = (32.1, 67.9).

Immune responses were summarized by the proportion of participants with a positive response to individual antigens at each time point, with boxplots showing the distributions of the immune response magnitudes among positive responders at each time point.

Barnard’s tests were used to compare immune response rates and Wilcoxon rank-sum tests to compare magnitudes among positive responders between the vaccine groups. Formal comparisons were not made for saturated binding antibody responses (i.e., median > 22,000 upper limit of quantitation). Two-sided 95% CIs for positive response rates were calculated using the Wilson (Miettinen-Nurminen) method [14].

All p-values are two-sided, with p-values less than 0.05 deemed statistically significant. For each distinct hypothesis, the number of multiple tests was limited, and therefore multiplicity adjustment was not done. SAS (version 9.4; SAS Institute, Cary, North Carolina) and R statistical software (version 4.0.4; R Foundation for Statistical Computing, Vienna, Austria) were used for statistical analyses.

The HVTN 100 protocol is available online: https://atlas.scharp.org/cpas/project/HVTN%20Public%20Data/HVTN%20100/begin.view.

## Results

### Participant cohort

In total, 70 participants of the 74 eligible HVTN 100 Part A participants were enrolled into HVTN 100 Part B: 32 eligible Part A vaccine recipients were randomized to Treatment (T) group T1a (ALVAC + gp120/MF59), 31 eligible Part A vaccine recipients were randomized to group T1b (gp120/MF59 alone) and 7 eligible Part A placebo recipients were assigned to Placebo (P) (Fig 1, S2 Table). Accrual into HVTN 100 Part B was from 13 September 2017 to 02 February 2018 and participants were followed for six months. The median age of participants enrolled was 25 years. There were 43 (61.4%) participants assigned male sex at birth and 27 (38.6%) participants assigned female sex at birth (Table 2).

**Table 2:** Participant demographics at enrollment.

	T1a (N = 32)	T1b (N = 31)	Placebo (N = 7)	Total (N = 70)
Sex assigned at birth, n (%)				
Male	20 (62.5%)	19 (61.3%)	4 (57.1%)	43 (61.4%)
Female	12 (37.5%)	12 (38.7%)	3 (42.9%)	27 (38.6%)
Race, n (%)				
Black	31 (96.9%)	31 (100.0%)	7 (100.0%)	69 (98.6%)
White	0 (0.0%)	0 (0.0%)	0 (0.0%)	0 (0.0%)
Color/Mixed	1 (3.1%)	0 (0.0%)	0 (0.0%)	1 (1.4%)
Age (years)				
Median	25	23	24	25
Range	19–38	19–32	19–39	19–39

### Safety results

Vaccinations were generally safe and well-tolerated with mostly mild to moderate local reactogenicity (S1 Fig). Among vaccine recipients, 6.4% (4/63) reported grade 2 erythema, 4.8% (3/63) reported grade 2 induration and 3.2% (2/63) reported grade 3 induration (≥10 cm diameter). Induration was on the right arm where gp120/MF59 was administered. There were no grade 4 local or systemic reactogenicities reported. Most systemic reactogenicity symptoms were mild: amongst participants randomized to ALVAC + gp120/MF59, 6.3% (2/32) reported moderate malaise and/or fatigue, 12.5% (4/32) myalgia, and 3.1% (1/32) arthralgia.

AEs were reported by 41.4% (29/70) of participants with 15 (48.4%) in participants randomized to gp120/ MF59 only, 13 (40.6%) in participants randomized to ALVAC + gp120/MF59 and 1 (14.3%) to a participant randomized to placebo and with no significant difference in AE reporting between the vaccine and placebo groups: 44.4% (28/63) vs 14.3% (1/7), p = 0.14. There were 4.8% (3/63) grade 3 elevated blood creatinine among both vaccine groups with 3.1% (1/32) in the ALVAC +gp120/MF59 group and 6.5% (2/31) in the gp120/MF59 group. These serum creatinine values remained within the local lab reference range throughout study, but, due to a particular feature of the adverse event grading table (increase from baseline value 2 years prior), were categorized as grade 3 AEs. The participants had no clinical evidence of adverse effects of the increase in creatinine and these AEs were assessed as not related to study agents. Two of the AEs resolved and the third was reported as ongoing at end of study. There were 17 (3 mild and 14 moderate) AEs due to infections and infestations, 9 (1 mild, 5 moderate and 3 severe) AEs due to abnormal investigation results, 4 mild renal and urinary disorder AEs, 1 mild back pain and 1 moderate skin ulcer, all assessed as not related to study product.

There were no serious AEs (SAEs), expedited AEs (EAEs), early terminations or discontinuations due to AEs, HIV acquisition, or cases of vaccine-induced seropositivity. Two pregnancies were reported, both from the gp120/MF59 group, with no congenital anomalies or adverse pregnancy outcomes reported.

### Immunogenicity results

#### HIV-1 binding antibody responses

It has been previously reported that the vaccine-induced binding IgG response rates and magnitudes were high at month 6.5 and month 12.5 (2 weeks after the fourth and fifth vaccinations, respectively) relative to placebo responses in Part A. Here, we report the vaccine-induced binding antibody responses to the month 30 (sixth) vaccination in the two Part B boost groups.

**Fig 2:**
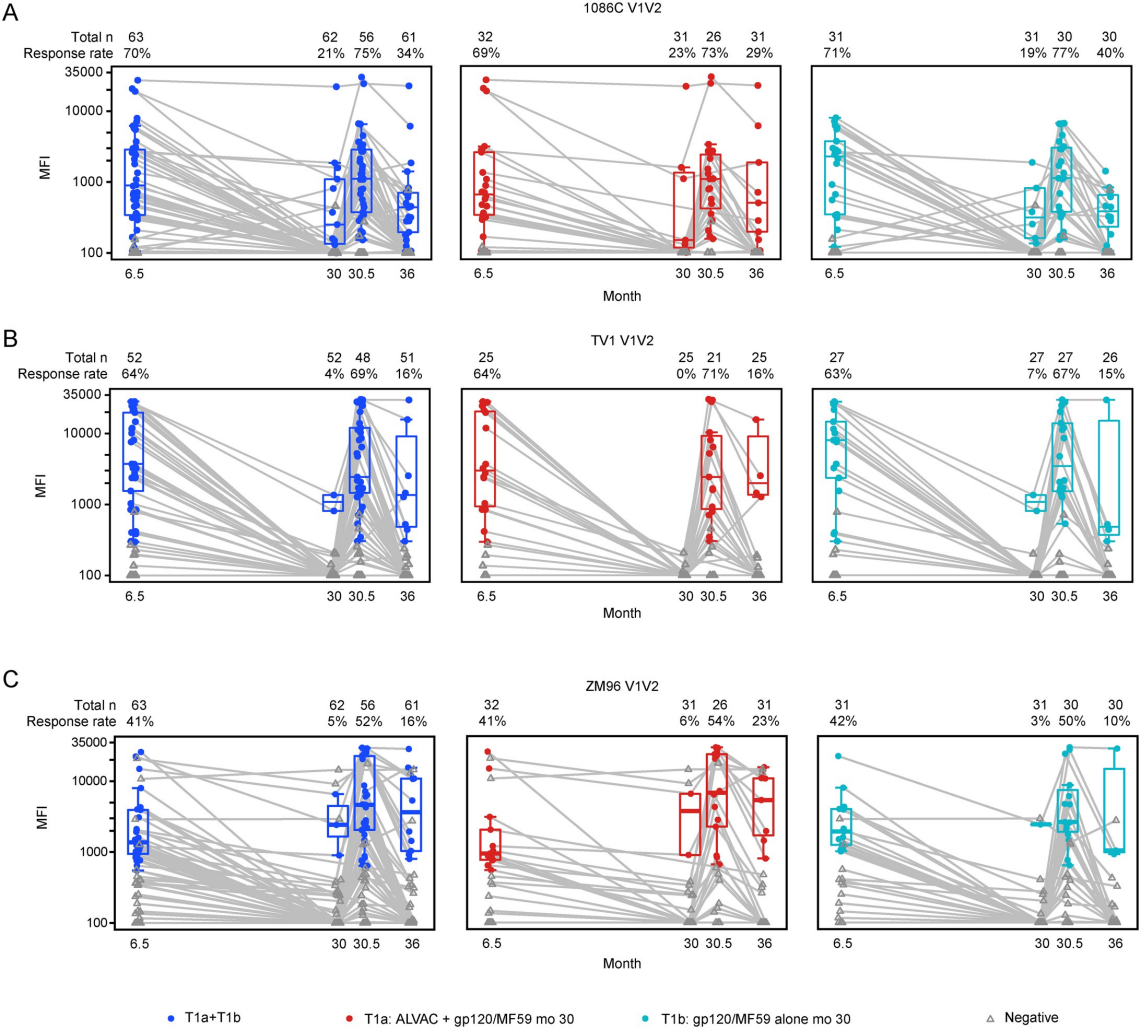
IgG binding antibody responses to the V1V2 vaccine strain antigens: 1086 (A), TV1 (B) and ZM 96 (C). Response rates and magnitudes of IgG binding antibodies to the V1V2 vaccine strain antigens: 1086 (A), TV1 (B) and ZM96 (C) among per-protocol vaccine recipients of HVTN 100 Part B. Boxplots show magnitude as mean fluorescent intensity (MFI) responses to individual V1V2 antigens and are based on positive responders, shown as solid circles, negative responders are shown as grey triangles. The box represents the interquartile range and distribution of data with the horizontal line in the box representing the median. The top and bottom whisker represents the maximum and minimum value that is not an outlier respectively.

In both the vaccine groups, positive binding IgG responses at month 30, month 30.5, and month 36 were observed to all V1V2 antigens (n = 27), to all gp120 antigens (n = 11) and to all gp140 antigens (n = 9) but were more common to gp120 antigens than to V1V2 antigens (Figs 2 and S2). There were no statistically significant differences between the 2 vaccine boost groups in binding IgG response rate or magnitude for any of the gp120, gp140 or V1V2 antigens at any of the late timepoints tested (months 30, 30.5 and 36).

Response rates and magnitudes two weeks after the month 30 boosts were similar to those seen at month 6.5 (Fig 2). There were low or no positive responses observed in the placebo boost group.

The binding IgG response rate at month 30 to gp120 antigens ranged between 6.5% (ZM96) to 98.4% (1086) for the two vaccine groups (S2 Fig). At month 30.5, positive binding IgG responses were observed in all 10 of the gp120 antigens that were tested, and the response rate ranged from 96.4% (ZM96) to 100% (all other antigens). At month 36, positive binding IgG response was observed in all 11 gp120 antigens tested with response rates ranging from 80.3% (ZM96) to 100% (1086, TV1, B6240, BORI and MN gp120 gDneg/293F).

The vaccine-induced binding antibody responses to subtype C gp120 antigens were higher for the 1086 and TV1 but lower for ZM96 for both vaccine groups with no significant difference between groups (S2 Fig). The binding IgG response rates to the month 30 booster vaccination were higher and more sustained until month 36 for gp120 (1086, ZM96 and TV1) (S2 Fig) compared to the response rates for the V1V2 antigens (1086 V1V2, TV1 V1V2, ZM96 V1V2) (Fig 2).

We also examined responses to two V1V2 antigens identified as correlates of risk in HVTN 702 (in conjunction with CD4+ T-cell responses) and in RV144, A244 V1V2 and B.CaseA V1V2, respectively. The IgG binding antibody responses to A244 V1V2 were similar at months 6.5 and 30.5 with waning by month 36 (S3A Fig), whereas responses to B.CaseA V1V2 appeared higher at month 30.5 than month 6.5 but also waned by month 36 (S3B Fig).

**Fig 3:**
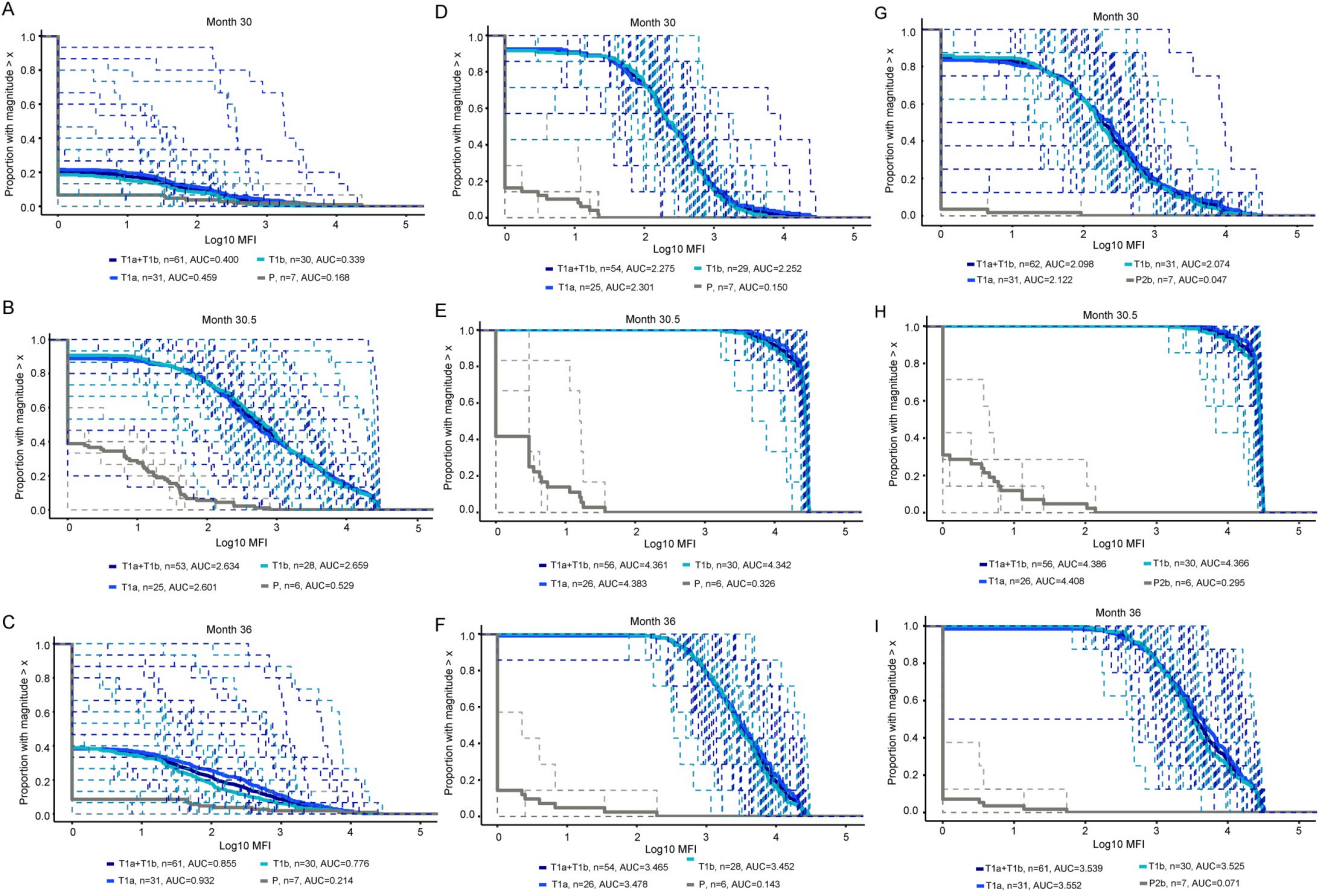
Magnitude-breadth of IgG binding antibody responses subtype C env V1V2 (A-C), gp120 (D-F) and gp140 (G-I) antigens among participants in the cohort for Part B at the Month 30 vaccination timepoint (A,D,G), 2 weeks after the Month 30 vaccination (Month 30.5) (B,E,H) and at Month 36 (C,F,I). Panel size is 15 (A-C), 7 (D,F), 6 (E), and 8 (G-I). AUC = area under curve, T = treatment, P = placebo, MFI = mean fluorescence intensity. T1a = ALVAC + gp120/MF59; T1b = gp120/MF49 alone.

Boosting of V1V2 responses did not result in as much breadth compared to gp120 and gp140 antigens. The month 30.5 magnitude-breadth IgG binding antibody responses to V1V2 antigens for the combined vaccine groups were considerably higher than the month 30 responses (median areas under the curve (AUCs) 2.55 vs. 0.13) but rapidly waned by month 36 (median AUC = 0.40) more strikingly than the gp120 or gp140 magnitude-breadth waning (Fig 3A–3C).

The magnitude-breadth IgG bAb responses to gp120 antigens for the combined vaccine groups were also higher at month 30.5 than month 30 (median AUCs 4.39 vs. 2.36), with waning by month 36 (median AUC = 3.47) (Fig 3D–3F). The month 30 vaccination also boosted the magnitude-breadth IgG bAb response to gp140 antigens at month 30.5 with a decline seen at month 36. Median AUCs for the combined vaccine groups was 2.10, 4.39 and 3.54 for months 30, 30.5 and 36, respectively (Fig 3G–3I).

#### Cellular immune responses

When comparing the two vaccine groups, recipients who received ALVAC + gp120/MF59 showed similar rates and similar magnitudes of Env-specific CD4+ T-cell responses at months 30.5 and 36, compared to recipients who received only gp120/MF59. However, there was one statistically significant difference in response magnitudes: higher Gag-specific IFN-γ and/or IL-2 CD4+ T-cell responses in the ALVAC + gp120/MF59 group compared to gp120/MF59 at month 30.5 (p = 0.0217). At the month 30.5 time-point, positive responses for CD4+ T-cell responses to the LAI Gag and CD8+ T-cell response rates to the vaccine-matched HIV peptide pools were low, observed in 4 or fewer participants (S4 and S5 Figs).

**Fig 4:**
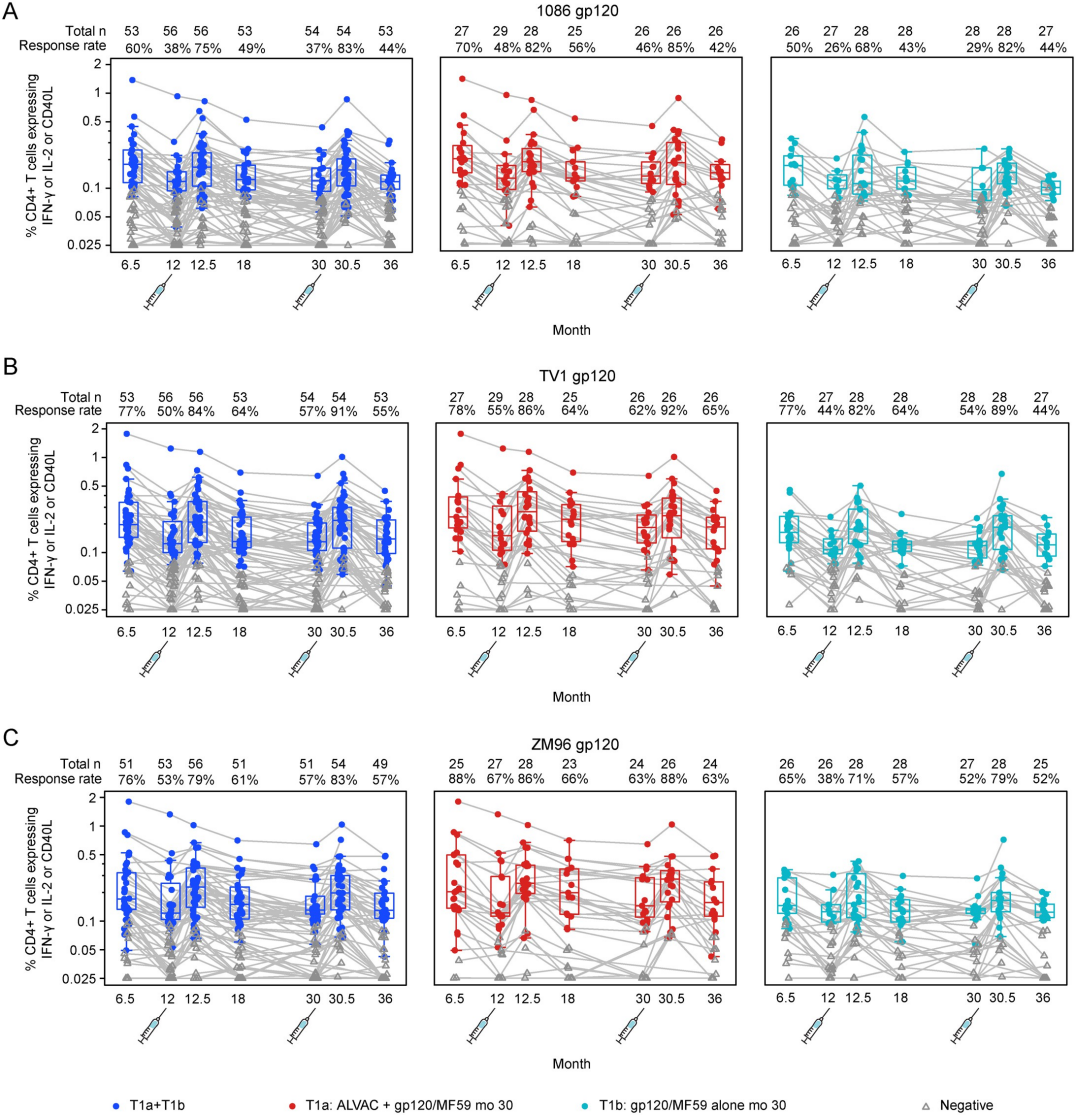
Summary of the response rates and magnitudes of CD4+ T cell responses. Boxplots show response magnitude in the percent expression of CD4+IFN-gamma and/or IL-2 and/or CD40L T cell responses to Env 1086 (A), TV1 (B) and ZM96 (C) and are based on positive responders, shown as solid circles, and negative responders are shown as grey triangles.

The CD4+ T-cell response rates to gp120 peptides were highest 2 weeks after vaccination (month 30.5), with significant expansion from month 30 to month 30.5 and subsequent contraction to month 36 observed after boosting and returned to similar response levels seen at month 12.5 (Fig 4).

#### ADCC responses

Positive ADCC luciferase responses were observed in the combined vaccine group at month 30.5 and month 36 with higher responses generally seen in the gp120/MF59 only group, with a significantly higher response rate seen at month 30.5 to TV1 (76.7% vs. 46.4%, p = 0.019). However, no significant difference was seen at month 36 and higher responses to TV1 seen at month 30.5 compared to earlier responses at month 12.5 (S6 Fig).

#### Neutralizing antibody responses

Neutralizing antibody responses were seen to tier 1A MW965.26 and TV1 over time, and were higher than responses seen at month 12.5, with no significant differences between vaccine groups at month 30.5 and 36 (S7 Fig). No tier 2 neutralizing responses were induced at any time.

## Discussion

The results of the HVTN 100 Part B study show that the addition of a late vaccine booster, even with an 18-month interval, showed an acceptable safety and reactogenicity profile, and restored immune responses temporarily. Durability of immune responses remains a challenge, with observed decay after 6 months. These findings may inform future HIV vaccination regimens requiring frequent vaccinations. Antibody waning may have contributed to the decline in protection seen 12 months post-vaccination in the RV144 trial. It is possible that establishing a protective antibody level for HIV prevention may better inform the timing of booster vaccination intervals. The additional adjuvanted booster response was disappointing. Continued homologous boosting seems ineffective in providing both antibody secreting cells and plasma cells (memory B cells were not analyzed). These data point out the need for different strategies to boost long term antibodies after vaccination.

The safety and tolerability of the month 30 boost was similar to earlier doses seen in the study [6,7]. The RV144 trial showed that HIV-1 acquisition risk was reduced with higher levels of total IgG and IgG3 binding antibodies to HIV Env and antibodies to scaffolded antigens, including the V1V2 loop [9,12,13]. There was also substantial heterogeneity among participants in the durability of the V1V2 antibody responses [11]. The current study showed that binding antibody responses to V1V2 may be restored by the addition of a late HIV vaccination, but maintaining these responses is a challenge. The extended vaccination regimen can only induce and boost neutralizing antibody responses to tier 1A viruses in magnitude and durability. These results are similar to RV305, a study where higher HIV-1 antigen-specific V1V2 and gp120 binding antibody titers were seen, and higher tier 1 neutralizing antibodies with booster vaccinations given between 6 to 8 years after the primary vaccination regimen [3]. Induction of tier 2 neutralizing antibodies has been challenging and may require a different vaccination strategy and improved antigen design. Neutralising antibodies were uncommon in RV144 vaccinees, so these responses likely did not contribute significantly to the modest HIV protection seen in the trial.

In the current study, the addition of ALVAC to the adjuvanted protein vaccine at month 30 did not enhance antibody responses compared to the group that received adjuvanted protein alone. Both vaccine regimens administered in HVTN 100 Part B at month 30, ALVAC-HIV + gp120/MF59 and gp120/MF59 alone, showed an acceptable safety and reactogenicity profile and induced HIV-specific antibody and cellular responses two weeks and 6 months after vaccination. However, there was no statistically significant difference between the two groups except for higher CD4+ Gag-specific cellular responses in the ALVAC-HIV + gp120/MF59 group, most likely attributed to the *gag* insert in the ALVAC-HIV component. The addition of ALVAC to the adjuvanted protein vaccine at month 30 did not enhance neutralizing antibody responses compared to the group that received adjuvanted protein alone.

This study does have some limitations. First, it included a small sample size (N = 70). Second, this study had a lower dose (100 micrograms) of gp120 compared to what was used in the RV144 study (300 micrograms) because it was thought that the change in the adjuvant from Alum to MF59 compensated for the reduced dose of gp120 [15–17]. Third, we were not able to assess ADCC and ADCP results compared to some of the earlier timepoints in HVTN 100 Part A due to changes in the assays used in HVTN 100 Part B.

Although the HVTN 702 trial demonstrated that ALVAC + gp120/MF59 did not prevent HIV acquisition, the results from our earlier study show that booster vaccines even after an 18-month vaccine-free interval are safe and can restore antibody and cellular responses. While the goal of an effective preventative HIV vaccine is to have high efficacy and durability of immune responses, it is encouraging that we are able to restore immune responses with delayed boosting. This finding may have relevance to booster strategies for other HIV vaccines and vaccines for other infectious diseases. The importance and effect of booster doses has been shown with other vaccines for infectious diseases, including the influenza and SARS-COV-2 vaccines.

## Supporting information

S1 Table Antigens used for the HVTN 100 Part B BAMA assays and ICS. (DOCX)

S2 Table Part B enrollment characteristics by Part A treatment assignment. (DOCX)

S1 Fig Stacked bar charts of maximum local and systemic reactogenicity over Month 30 vaccination. Local (A) and systemic (B) reactogenicity events. P-values indicate differences between any of the vaccine and placebo groups (T1a, T1b, P). P = placebo. T1a (ALVAC + gp120/MF59); T1b (gp120/MF59 alone). There were no Grade 4/complications/life-threatening events.
(TIF)

S2 Fig IgG binding antibody responses to gp120 antigens 1086, TV1 and ZM96. Y axis is mean fluorescent intensity and x axis is treatment group distributed by study month. Month 30 is time of vaccination, month 30.5 is two weeks post-vaccination and month 36 is 6 months post-vaccination. A: 1086 gp120; B: TVI gp120; C: ZM96 gp120. Red dots are T1a (ALVAC + gp120/MF59), turquoise dots are T1b (gp120/MF59 alone), and blue dots are T1a+T1b. Participants without a response are gray triangles. The box represents the interquartile range and distribution of data with the horizontal line in the box representing the median. The top and bottom whisker represents the maximum and minimum value that is not an outlier respectively.
(TIF)

S3 Fig V1V2 BAMA responses 702 and RV144 V1V2 correlates. Y axis is mean fluorescent intensity (MFI), and x axis is month post first vaccination. Each dot or triangle is one participant. Red dots are T1a (ALVAC + gp120/MF59), turquoise dots are T1b (gp120/MF59 alone), and blue dots are T1a+T1b. Participants without a response are gray triangles. The box represents the interquartile range and distribution of data with the horizontal line in the box representing the median. The top and bottom whisker represents the maximum and minimum value that is not an outlier respectively.
(TIF)

S4 Fig CD4+ T-cell response rates and magnitudes to Gag by ICS. Y axis is percentage of CD4+ T cells expressing IFN-γ or IL-2 or CD40L, and x axis is month post first vaccination. Syringes denote timepoint of vaccination. Each dot or triangle is one participant. Red dots are T1a (ALVAC + gp120/MF59), turquoise dots are T1b (gp120/MF59 alone), and blue dots are T1a+T1b. Participants without a response are gray triangles. The box represents the interquartile range and distribution of data with the horizontal line in the box representing the median. The top and bottom whisker represents the maximum and minimum value that is not an outlier respectively.
(TIF)

S5 Fig CD8+ T-cell response rates and magnitudes to any HIV. Y axis is percentage of CD8+ T cells expressing IFN-γ or IL-2 or CD40L, and x axis is month post first vaccination. Each dot or triangle is one participant. Red dots are T1a (ALVAC + gp120/MF59), turquoise dots are T1b (gp120/MF59 alone), and blue dots are T1a+T1b. Participants without a response are gray triangles. The box represents the interquartile range and distribution of data with the horizontal line in the box representing the median. The top and bottom whisker represents the maximum and minimum value that is not an outlier respectively. A) 1086C gp120, B) TV1 gp120, C) ZM96 gp120, D) LAI Gag.
(TIF)

S6 Fig ADCC luciferase pAUC to vaccine matched antigens. Summary of response rates and magnitudes of antibody-dependent cell-mediated cytotoxicity luciferase to 1086 (A), TV1 (B) and ZM96 (C). Boxplots show response magnitude in the partial area under curve baseline-subtracted percentage loss activity to each antigen and are based on positive responders, shown as solid circles, and negative responders are shown as grey triangles. The box represents the interquartile range and distribution of data with the horizontal line in the box representing the median. The top and bottom whisker represents the maximum and minimum value that is not an outlier respectively.
(TIF)

S7 Fig Summary of response rates and magnitudes to tier 1A MW965.26.C and TV1 among vaccine recipients of HVTN 100 Part B. Boxplots show response magnitude as the ID50 neutralizing antibody titer and are based on positive responders, shown as solid circles, negative responders are shown as grey triangles. The box represents the interquartile range and distribution of data with the horizontal line in the box representing the median. The top and bottom whisker represents the maximum and minimum value that is not an outlier respectively.
(TIF)

S1 Text (DOCX)
