# Supplementary material for: Safety and immunogenicity after a 30-month boost of a subtype C ALVAC-HIV (vCP2438) vaccine prime plus bivalent subtype C gp120/MF59 vaccine boost (HVTN 100): A phase 1–2 randomized double-blind placebo-controlled trial
Source: PLOS Glob Public Health. 2024 Sep 20;4(9):e0003319. doi: 10.1371/journal.pgph.0003319 (PMC11414935; doi:10.1371/journal.pgph.0003319)
Supplement: S1 Table — (DOCX) [file pgph.0003319.s001.docx]

| **Table S1. Antigens used for the HVTN 100 Part B BAMA assays and ICS** | | | |
| --- | --- | --- | --- |
| **Antigen class** | **Antigen** | **Subtype** | **Antigen label used in plots** |
| **BAMA bAb** | | | |
| gp41 | gp41 | B |  |
|  | MN gp120 gDneg/293F | B |  |
|  | Con S gp140 CFI | Group M consensus |  |
| gp120 vaccine strain panel | 1086C_D7gp120.avi/293F | C | 1086 gp120 |
|  | TV1c8_D11gp120.avi/293F | C | TV1 gp120 |
|  | 96ZM651.D11gp120.avi | C | ZM96 gp120 |
| gp140 breadth panel | 9004S.gp140C.avi | A |  |
|  | RHPA4259_C7.gp140C.avi | B |  |
|  | SC42261_gp140.avi/293F | B |  |
|  | WITO4160.gp140C.avi | B |  |
|  | BF1266_gp140C.avi/293F | C |  |
|  | C.CH505TF_gp140/293F | C (Data missing for visit 19) |  |
|  | 1086C gp140C_avi | C |  |
| gp120 breadth panel | 51802_D11gp120.avi/293F | A |  |
|  | 254008_D11gp120.avi/293F | CRF01_AE (not included in analysis) |  |
|  | TT31P.2792_D11gp120.avi/293F | B |  |
|  | B.6240_D11gp120/293F | B | B.6240 |
|  | BORI_D11gp120.avi/293F | B (Data missing for visit 19) | BORI |
|  | A244 D11gp120_avi | CRF01-AE |  |
|  | CNE20_D11gp120.avi/293F | CRF07-BC |  |
|  | BJOX002_D11gp120.avi/293F | CRF07-BC |  |
| gp70 V1V2 breadth panel | gp70-191084_B7 V1V2 | A |  |
|  | gp70_B.CaseA_V1_V2 | B | B.CaseA V1V2 |
|  | gp70-RHPA4259.7 V1V2 | B |  |
|  | gp70-62357.14 V1V2 | B |  |
|  | gp70-700010058 V1V2 | B |  |
|  | gp70-TT31P.2F10.2792 V1V2 | B (Data missing for visits 17 and 20) |  |
|  | gp70-BF1266_431a_V1V2 | C |  |
|  | gp70-7060101641 V1V2 | C |  |
|  | gp70-96ZM651.02 V1V2 | C | ZM96 V1V2 |
|  | gp70-001428.2.42 V1V2 | C |  |
|  | gp70-CAP210.2.00.E8 V1V2 | C |  |
|  | gp70-TV1.21 V1V2 | C | TV1 V1V2 |
|  | gp70-C.1086C V1/V2/293F | C |  |
|  | gp70-CM244.ec1 V1V2 | CRF01-AE |  |
|  | gp70-C2101.c01_V1V2 | CRF01-AE |  |
|  | gp70-BJOX002000.03.2 V1V2 | CRF07-BC |  |
| Additional vaccine strain gp70 V1V2 antigens | C.1086C_V1_V2 Tags | C | 1086 V1V2 |
|  | gp70-TV1.GSKvacV1V2/293F | C | TV1.gsk V1V2 |
| Additional gp70 V1V2 Antigens | AE.A244 V1V2 Tags/293F | AE | A244 V1V2 |
|  | gp70 B.CaseA2 V1/V2/169K | B | B.CaseA V1V2 |
|  | gp70-1012.11.TC21.3257 V1V2 | C |  |
|  | gp70-1051.12.C22 V1V2 | B |  |
|  | gp70-1394C9G1 V1V2 | C |  |
|  | gp70-CAP45.2.00.G3 V1V2 | C |  |
|  | gp70-Ce1176 V1V2 | C |  |
|  | gp70-Ce704010042_2ES V1V2 | C |  |
|  | gp70-ConC V1V2 | C |  |
|  | gp70-Du156.12 V1V2 | C |  |
| **ICS** | | | |
| gp120 | 1086.C gp120 | C | 1086 gp120 |
|  | TV1 gp120 | C | TV1 gp120 |
| gp140 | ZM96 gp140 | C | ZM96 gp140 |
| **ADCC** | | | |
| gp120 | 1086 gp120 | C | 1086 gp120 |
|  | TV1 gp120 | C | TV1 gp120 |
|  | ZM96 gp140 | C | ZM96 gp140 |
