# Supplementary material for: Safety and immunogenicity after a 30-month boost of a subtype C ALVAC-HIV (vCP2438) vaccine prime plus bivalent subtype C gp120/MF59 vaccine boost (HVTN 100): A phase 1–2 randomized double-blind placebo-controlled trial
Source: PLOS Glob Public Health. 2024 Sep 20;4(9):e0003319. doi: 10.1371/journal.pgph.0003319 (PMC11414935; doi:10.1371/journal.pgph.0003319)
Supplement: S2 Table — (DOCX) [file pgph.0003319.s002.docx]

| **Table S2. Part B enrollment characteristics by Part A treatment assignment.** | | | |
| --- | --- | --- | --- |
|  | P  (N=17)  n (%) | T1  (N=118)  n (%) | Total  (N=135)  n (%) |
| Participants eligible and willing to continue in Part B | | | |
| Yes | 7 (41.2%) | 67 (56.8%) | 74 (54.8%) |
| No | 10 (58.8%) | 51 (43.2%) | 61 (45.2%) |
| If ineligible/unwilling, primary reason for not joining Part B | | | |
| Co-enrollment in a study with an investigational research agent | 0 (0%) | 0 (0%) | 0 (0%) |
| Clinically significant condition | 1 (5.9%) | 6 (5.1%) | 7 (5.2%) |
| Participant death | 0 (0%) | 0 (0%) | 0 (0%) |
| Investigator decision | 1 (5.9%) | 12 (10.2%) | 13 (9.6%) |
| Participant refused further participation | 1 (5.9%) | 2 (1.7%) | 3 (2.2%) |
| Participant relocated | 0 (0%) | 1 (0.8%) | 1 (0.7%) |
| Participant lost to follow-up | 1 (5.9%) | 4 (3.4%) | 5 (3.7%) |
| HIV infection | 2 (11.8%) | 0 (0%) | 2 (1.5%) |
| Unwilling to provide mucosal secretion sample | 0 (0%) | 2 (1.7%) | 2 (1.5%) |
| Other, specify | 4 (23.5%) | 24 (20.3%) | 28* (20.7%) |
| Total | 10 (58/8%) | 51 (43.2%) | 61 (45.2%) |
| *~14 participants were unable to enroll within the visit window, 7 did not meet the eligibility criteria related to pregnancy, breastfeeding, and contraception, and some were unable to adhere to the Part B visit schedule. | | | |
