# Supplementary figures and images for: Safety and immunogenicity after a 30-month boost of a subtype C ALVAC-HIV (vCP2438) vaccine prime plus bivalent subtype C gp120/MF59 vaccine boost (HVTN 100): A phase 1–2 randomized double-blind placebo-controlled trial
Source: PLOS Glob Public Health. 2024 Sep 20;4(9):e0003319. doi: 10.1371/journal.pgph.0003319 (PMC11414935; doi:10.1371/journal.pgph.0003319)

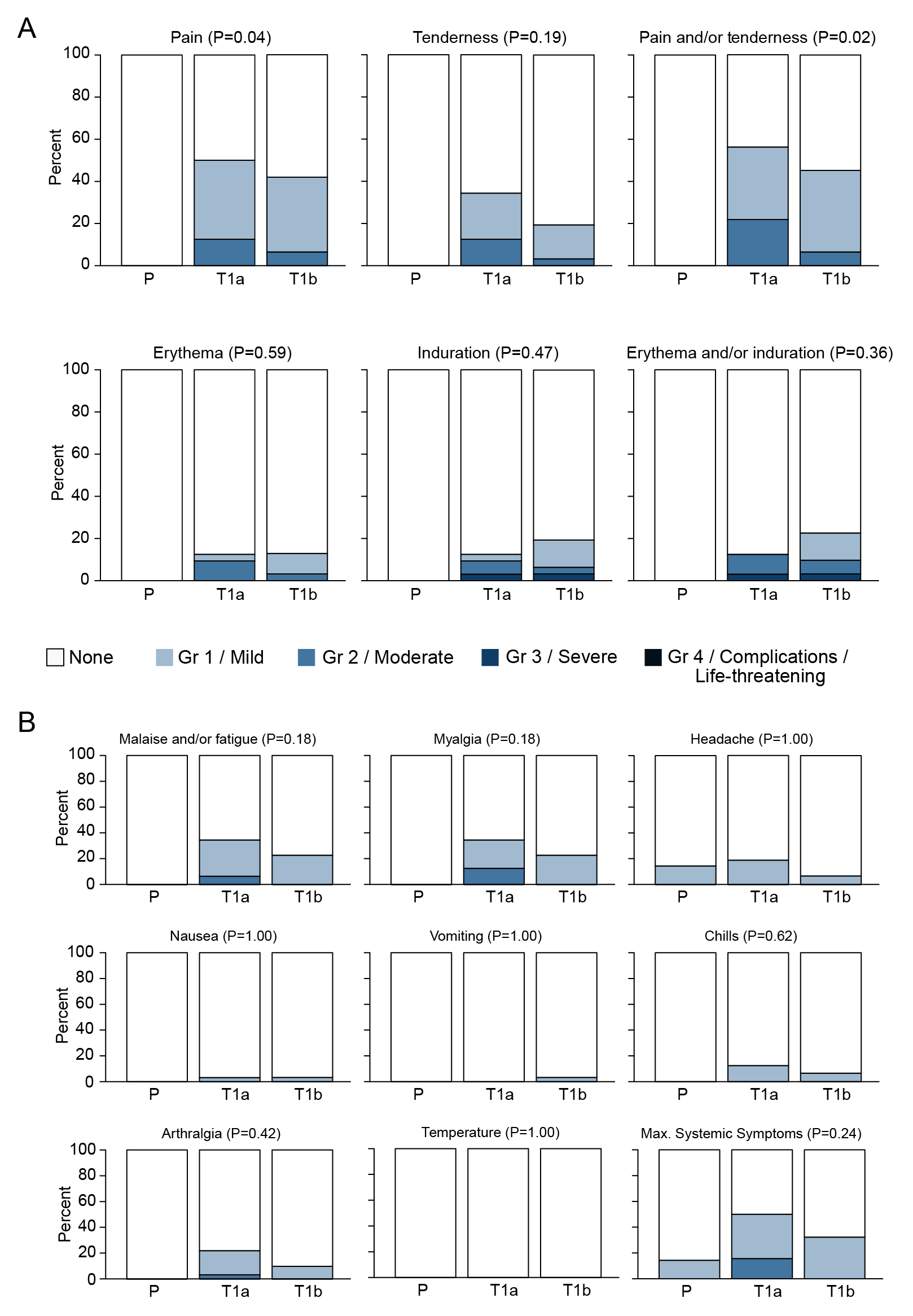

Supplement: S1 Fig — Local (A) and systemic (B) reactogenicity events. P-values indicate differences between any of the vaccine and placebo groups (T1a, T1b, P). P = placebo. T1a (ALVAC + gp120/MF59); T1b (gp120/MF59 alone). There were no Grade 4/complications/life-threatening events. (TIF) [file pgph.0003319.s003.tif]

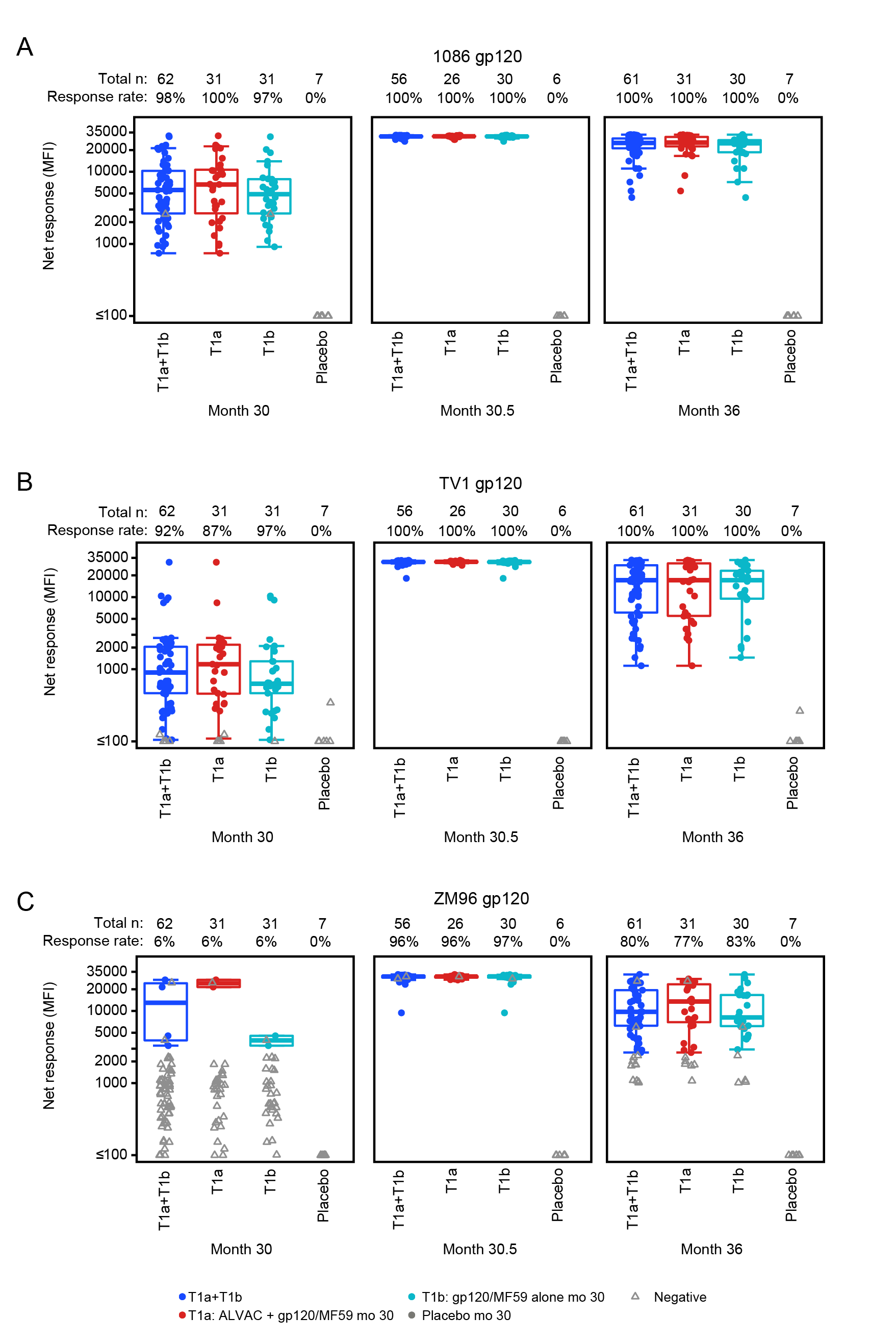

Supplement: S2 Fig — Y axis is mean fluorescent intensity and x axis is treatment group distributed by study month. Month 30 is time of vaccination, month 30.5 is two weeks post-vaccination and month 36 is 6 months post-vaccination. A: 1086 gp120; B: TVI gp120; C: ZM96 gp120. Red dots are T1a (ALVAC + gp120/MF59), turquoise dots are T1b (gp120/MF59 alone), and blue dots are T1a+T1b. Participants without a response are gray triangles. The box represents the interquartile range and distribution of data with the horizontal line in the box representing the median. The top and bottom whisker represents the maximum and minimum value that is not an outlier respectively. (TIF) [file pgph.0003319.s004.tif]

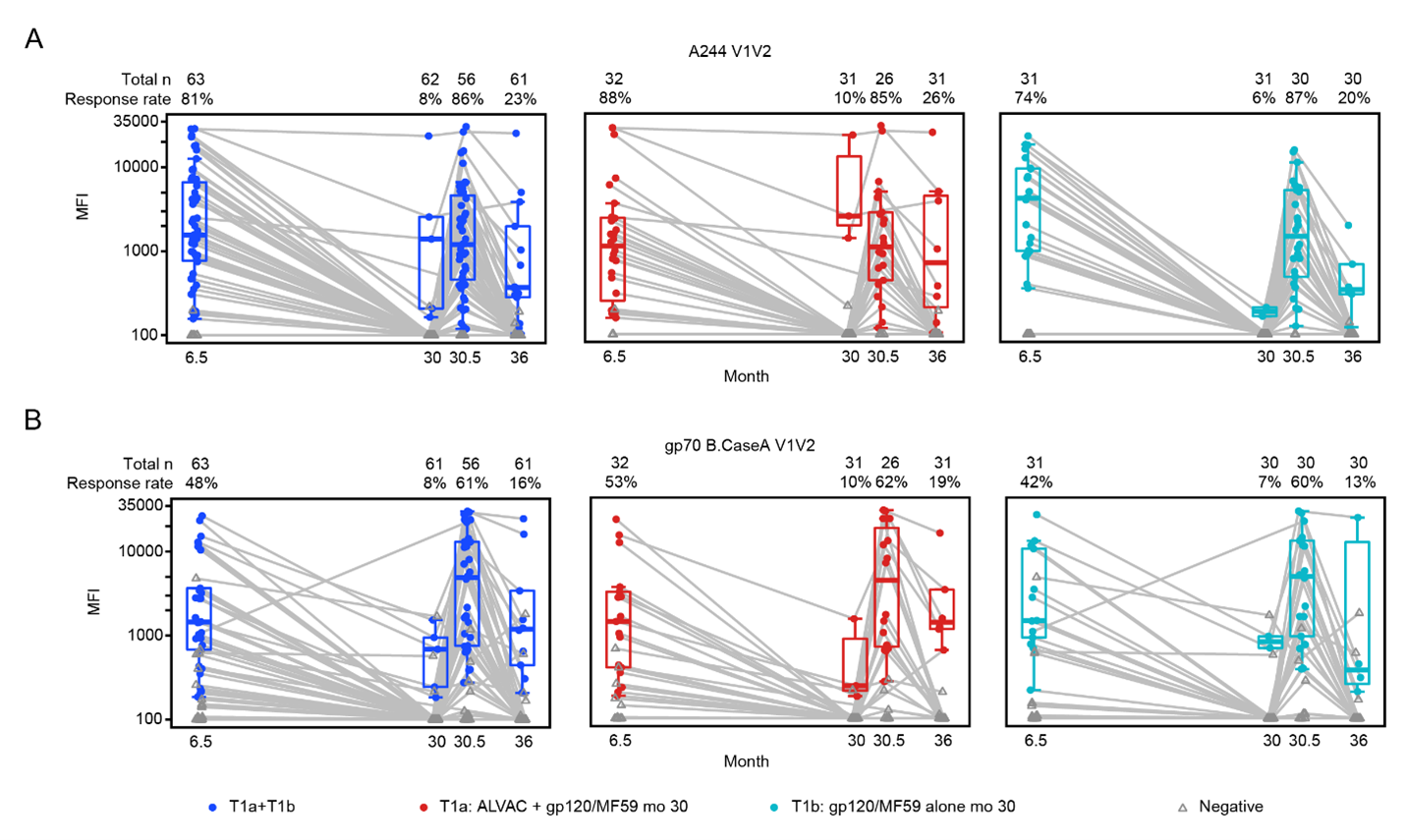

Supplement: S3 Fig — Y axis is mean fluorescent intensity (MFI), and x axis is month post first vaccination. Each dot or triangle is one participant. Red dots are T1a (ALVAC + gp120/MF59), turquoise dots are T1b (gp120/MF59 alone), and blue dots are T1a+T1b. Participants without a response are gray triangles. The box represents the interquartile range and distribution of data with the horizontal line in the box representing the median. The top and bottom whisker represents the maximum and minimum value that is not an outlier respectively. (TIF) [file pgph.0003319.s005.tif]

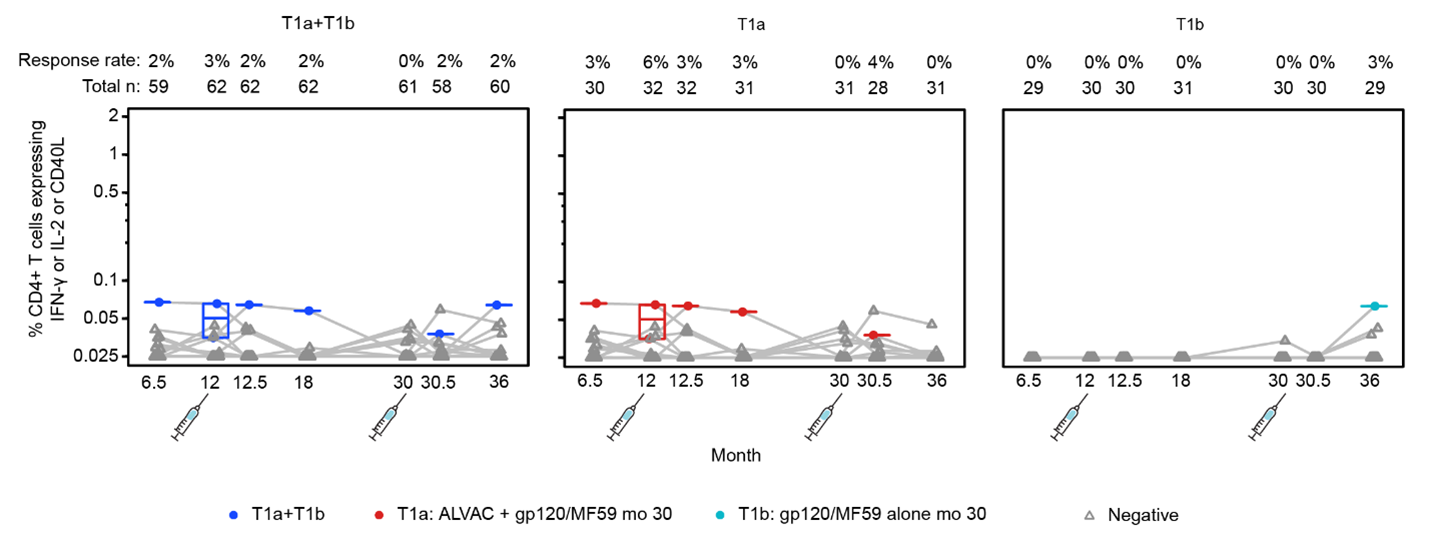

Supplement: S4 Fig — Y axis is percentage of CD4+ T cells expressing IFN-γ or IL-2 or CD40L, and x axis is month post first vaccination. Syringes denote timepoint of vaccination. Each dot or triangle is one participant. Red dots are T1a (ALVAC + gp120/MF59), turquoise dots are T1b (gp120/MF59 alone), and blue dots are T1a+T1b. Participants without a response are gray triangles. The box represents the interquartile range and distribution of data with the horizontal line in the box representing the median. The top and bottom whisker represents the maximum and minimum value that is not an outlier respectively. (TIF) [file pgph.0003319.s006.tif]

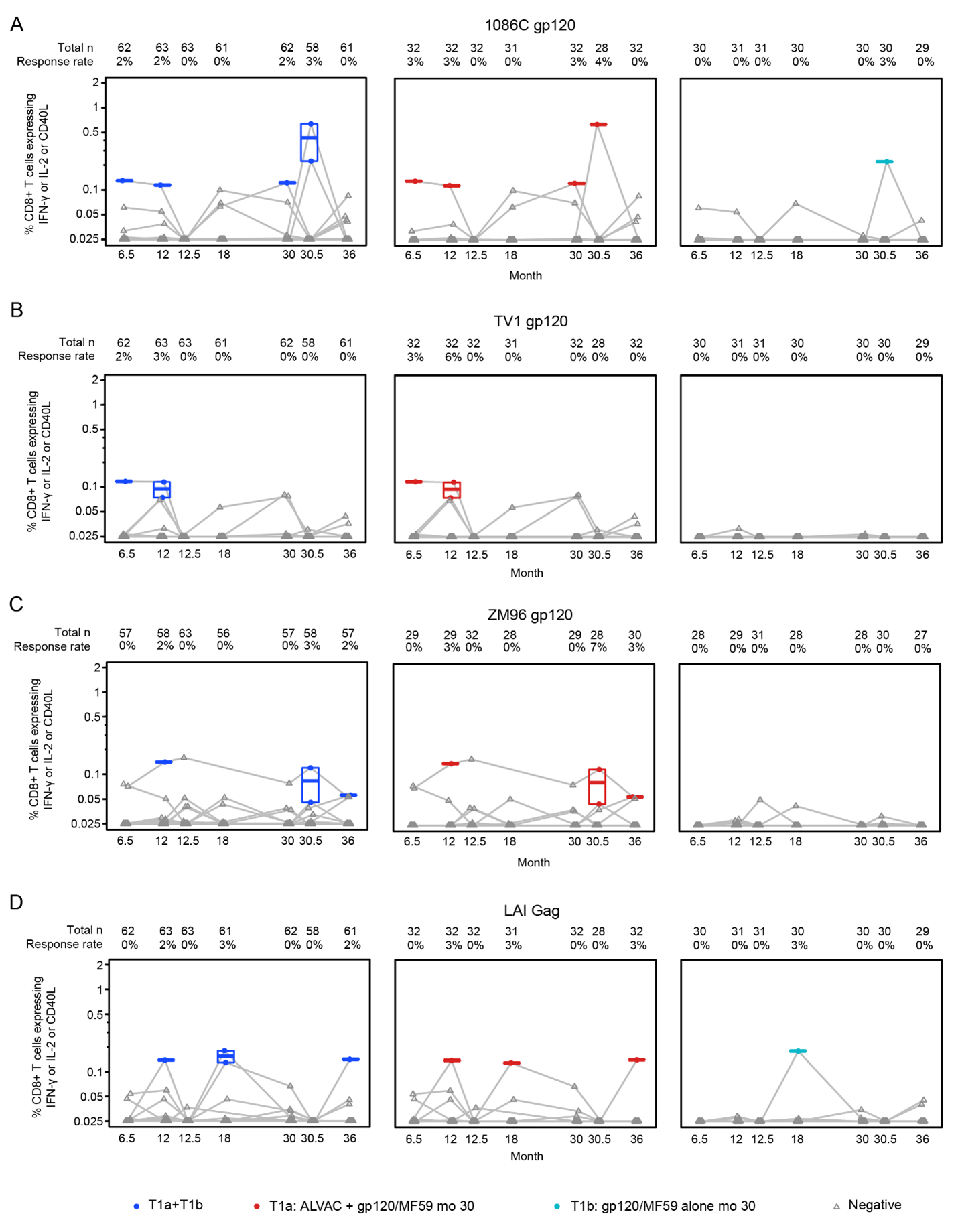

Supplement: S5 Fig — Y axis is percentage of CD8+ T cells expressing IFN-γ or IL-2 or CD40L, and x axis is month post first vaccination. Each dot or triangle is one participant. Red dots are T1a (ALVAC + gp120/MF59), turquoise dots are T1b (gp120/MF59 alone), and blue dots are T1a+T1b. Participants without a response are gray triangles. The box represents the interquartile range and distribution of data with the horizontal line in the box representing the median. The top and bottom whisker represents the maximum and minimum value that is not an outlier respectively. A) 1086C gp120, B) TV1 gp120, C) ZM96 gp120, D) LAI Gag. (TIF) [file pgph.0003319.s007.tif]

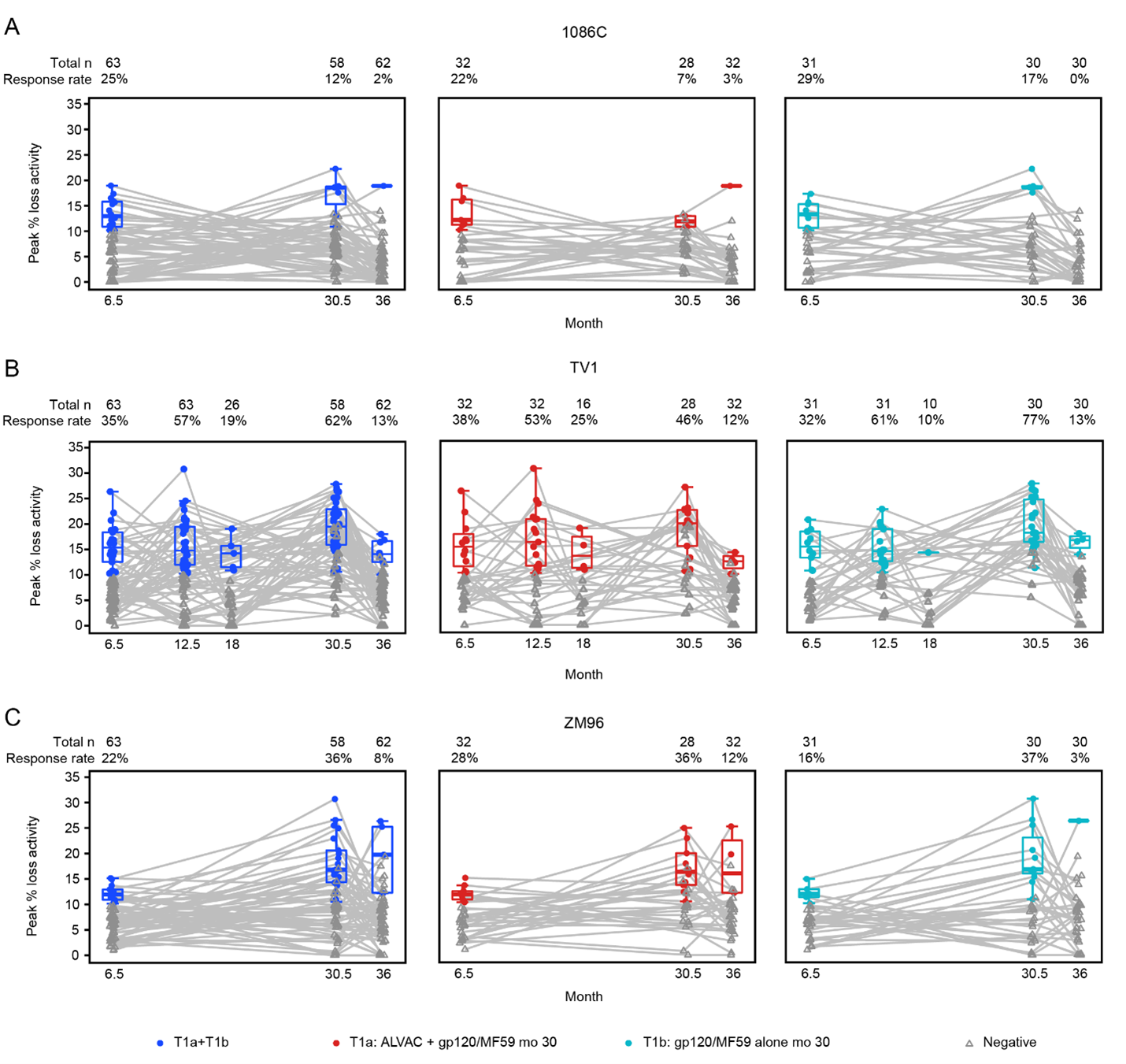

Supplement: S6 Fig — Summary of response rates and magnitudes of antibody-dependent cell-mediated cytotoxicity luciferase to 1086 (A), TV1 (B) and ZM96 (C). Boxplots show response magnitude in the partial area under curve baseline-subtracted percentage loss activity to each antigen and are based on positive responders, shown as solid circles, and negative responders are shown as grey triangles. The box represents the interquartile range and distribution of data with the horizontal line in the box representing the median. The top and bottom whisker represents the maximum and minimum value that is not an outlier respectively. (TIF) [file pgph.0003319.s008.tif]

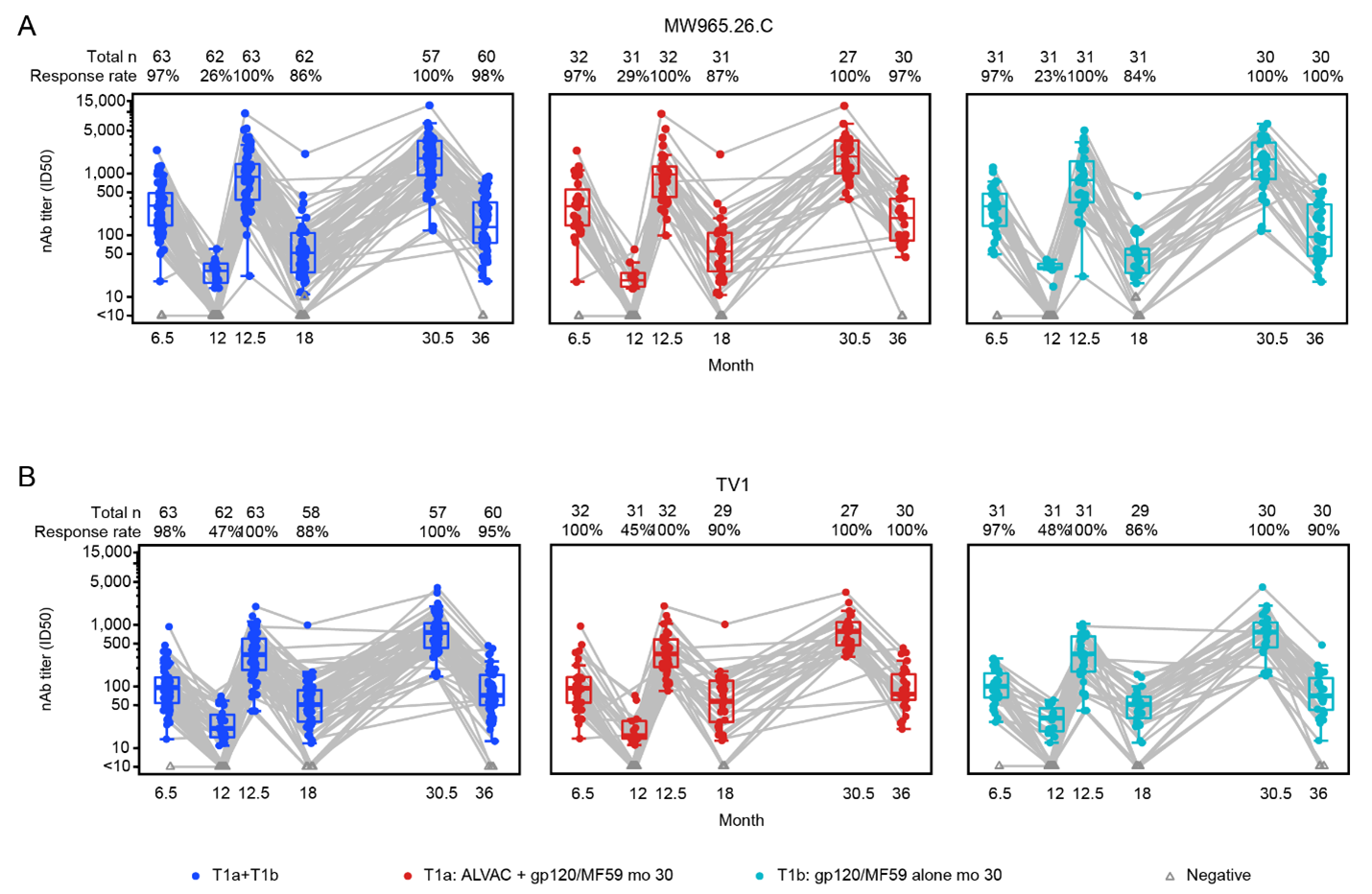

Supplement: S7 Fig — Boxplots show response magnitude as the ID50 neutralizing antibody titer and are based on positive responders, shown as solid circles, negative responders are shown as grey triangles. The box represents the interquartile range and distribution of data with the horizontal line in the box representing the median. The top and bottom whisker represents the maximum and minimum value that is not an outlier respectively. (TIF) [file pgph.0003319.s009.tif]
