# Supplementary material for: Safety and immunogenicity after a 30-month boost of a subtype C ALVAC-HIV (vCP2438) vaccine prime plus bivalent subtype C gp120/MF59 vaccine boost (HVTN 100): A phase 1–2 randomized double-blind placebo-controlled trial
Source: PLOS Glob Public Health. 2024 Sep 20;4(9):e0003319. doi: 10.1371/journal.pgph.0003319 (PMC11414935; doi:10.1371/journal.pgph.0003319)
Supplement: S1 Text — (DOCX) [file pgph.0003319.s010.docx]

**S1 Text**

**Antibody-dependent cell-mediated cytotoxicity (ADCC)**

ADCC-mediated antibody responses were measured using GranToxiLux (GTL) assays and luciferase from specimens obtained at months 30, 30.5 and 36, corresponding to the 6^th^ vaccination, 2 weeks after the 6^th^ vaccination and 6 months after the 6^th^ vaccination.

The GTL ADCC assay measures percent granzyme B activity, defined as the percentage of antigen coated target cells positive for proteolytically active Granzyme B out of the total viable target cell population. Endpoints are the response rate and magnitude of ADCC-mediated antibody responses against 3 HIV-1 antigens ZM96 gp120, 1086.C gp120 and TV1 gp120 using gp120 coated cells. Target cells were a clonal isolate of the CEM.NKRCCR5 CD4+ T-cell line coated with recombinant gp120s representing the HIV-1 envelopes of the subtype. Effector cells were PBMC obtained from the HIV negative donor. PBMC used as an effector cell to target cell ratio 30:1. Serum samples were tested after 5-fold dilutions starting at 1:50. Flow cytometry was used to quantify the frequency of granzyme B positive cells. ADCC was quantified as the net percent granzyme B activity percentage target cells positive for GTL (indicator of granzyme B uptake) – percentage of target cells positive for GTL when incubated with effector cells in the absence of any source antibodies. Granzyme B activity was measured at 6 dilution levels: 50, 250, 1250, 6250, 31250 and 156250 for each antigen. Positive peak activity was ≥8%.

A modified version of the previously published ADCC luciferase procedure was used in this study. The targets for ADCC luciferase assay after infection by an HIV-1 infectious molecular clone were CEM.NKRCCR5 cells. Cryopreserved PBMCs were obtained from an HIV-negative donor which were thawed and rested overnight for the assay, supplemented with antibiotics, 10% fetal bovine serum and 10 ng/ml IL-15 before use as effector cells at a ratio of 30:1.
